# Necessity of prophylactic splenic hilum lymph node clearance for middle and upper third gastric cancer: a network meta-analysis

**DOI:** 10.1186/s12885-020-6619-8

**Published:** 2020-02-24

**Authors:** Gaozan Zheng, Jinqiang Liu, Yinghao Guo, Fei Wang, Shushang Liu, Guanghui Xu, Man Guo, Xiao Lian, Hongwei Zhang, Fan Feng

**Affiliations:** 1 Department of Digestive Surgery, Xijing Hospital of Digestive Diseases, the Fourth Military Medical University, 127 West Changle Road, Xi’an, 710032 Shaanxi China; 2 Cadre’ s sanitarium, 62101 Army of PLA, 67 Nahu Road, Xinyang, 464000 Henan China; 3 Health company, 92667 Army of PLA, 39 East Zaoshan Road, Qingdao, 266100 Shandong China; 4 Department of General Surgery, No. 534 Hospital of PLA, West Lichun Road, Luoyang, 471000 Henan China

**Keywords:** Gastric cancer, Splenic hilar, Lymph node, Splenectomy, Spleen-preserving

## Abstract

**Background:**

It remains controversial whether prophylactic No.10 lymph node clearance is necessary for gastric cancer. Thus, the present study aims to investigate the impact of prophylactic No.10 lymph node clearance on the perioperative complications and prognosis of upper and middle third gastric cancer.

**Methods:**

A network meta-analysis to identify both direct and indirect evidence with respect to the comparison of gastrectomy alone (G-A), gastrectomy combination with splenectomy (G + S) and gastrectomy combination with spleen-preserving splenic hilar dissection (G + SPSHD) was conducted. We searched Medline, Embase, and the Cochrane Central Register of Controlled Trials (CENTRAL) for studies published before September 2018. Perioperative complications and overall survival were analyzed. Hazard ratios (HR) were extracted from the publications on the basis of reported values or were extracted from survival curves by established methods.

**Results:**

Ten retrospective studies involving 2565 patients were included. In the direct comparison analyses, G-A showed comparable 5-year overall survival rate (HR: 1.1, 95%CI: 0.97–1.3) but lower total complication rate (OR: 0.37, 95%CI: 0.17–0.77) compared with G + S. Similarly, the 5-year overall survival rate between G + SPSHD and G + S was comparable (HR: 1.1, 95%CI: 0.92–1.4), while the total complication rate of G + SPSHD was lower than that of G + S (OR: 0.50, 95%CI: 0.28–0.88). In the indirect comparison analyses, both the 5-year overall survival rate (HR: 1.0, 95%CI: 0.78–1.3) and total complication rate (OR: 0.75, 95%CI: 0.29–1.9) were comparable between G-A and G + SPSHD.

**Conclusions:**

Prophylactic No.10 lymph node clearance was not recommended for treatment of upper and middle third gastric cancer.

## Background

Gastric cancer remains the second most common cause of cancer-related death worldwide despite a decline in incidence [1]. Surgery is the main treatment for patients with gastric cancer. Even when surgery is combined with perioperative chemotherapy and/or radiation, outcomes remain poor. Therefore, various perioperative treatment strategies have been investigated to improve the surgical outcomes. Previous studies reported that D2 lymphadenectomy could achieve curability and prolong survival of gastric cancer [2]. Furthermore, it is generally accepted that gastrectomy with more lymph nodes resected could decrease recurrence rates and improve survival of gastric cancer patients [3]. While extended lymphadenectomy is associated with improved locoregional disease control, decreased recurrence rates, and improved DSS, it seems inappropriate to claim D2 lymphadenectomy could “achieve curability.”

The frequency of lymph node metastasis (LNM) to the splenic hilum and splenic artery has been reported as 10–20% in upper and middle third gastric cancer [4, 5], and the 5-year overall survival rate of patents with splenic hilar lymph node metastasis (SHLNM) was significantly lower than those without SHLNM [6–9]. Therefore, it is necessary to dissect No.10 lymph node by splenectomy or spleen-preserving splenic hilar dissection (SPSHD) for patients with evident macroscopic enlarged lymph node at the splenic hilar. However, for patients without enlarged splenic hilar lymph node, the value of prophylactic No.10 clearance for the prognosis of gastric cancer patients remains controversial. A series of studies emphasized the necessity of lymph node dissection at the splenic hilum to remove the potentially affected lymph nodes [10–13]. However, some investigators reported that No.10 lymphadenectomy was not associated with improved oncologic outcomes but was only associated with increased rates of postoperative complications [14–16].

Thus, the aim of this study was to evaluate the impact of prophylactic No.10 lymph node clearance on the perioperative complications and prognosis of middle and upper third gastric cancer.

## Methods

### Search strategy and selection criteria

For this network meta-analysis, Two investigators (Z.G and L.J) searched PubMed, the Cochrane Central Register of Controlled Trials (CENTRAL) and Embase for both retrospective study and randomized controlled trials (RCTs) published from the date of database inception to September 2018, of which the search strings were based on MeSH terms: “Stomach Neoplasms [mesh] OR Neoplasm, Stomach [title] OR Stomach Neoplasm [title] OR Neoplasms, Stomach [title] OR Gastric Neoplasms [title] OR Gastric Neoplasm [title] OR Neoplasm, Gastric [title] OR Neoplasms, Gastric [title] OR Cancer of Stomach [title] OR Stomach Cancers [title] OR Gastric Cancer [title] OR Cancer, Gastric [title] OR Cancers, Gastric [title] OR Gastric Cancers [title] OR Stomach Cancer [title] OR Cancer, Stomach [title] OR Cancers, Stomach [title] OR Cancer of the Stomach [title] OR Gastric Cancer, Familial Diffuse [title] OR Gastrectomies [title] OR Gastrectomy [title] OR gastric resection [title]) AND (Splenectomy [mesh] OR Splenectomies [title] OR spleen dissection [title] OR spleen-preserving [title] OR splenic preservation [title] OR Spleen preservation [title] OR preserving spleen [title] OR Spleen conserving [title] OR reserving Spleen [title] OR spleen preserving [title] OR Spleen-conserving [title] OR Splenic hilar [title] OR splenichilus [title] OR splenic hilum [title] OR hilum of spleen [title] OR No.10[title] OR No. 10[title]”. Additionally, we reviewed the reference lists of all the meta-analyses.

Studies with the following situations were considered excluded: (1) Studies with other kinds of gastric tumors, such as lymphoma, other organ tumors or multiple gastric tumors; (2) Studies with splenectomy induced by iatrogenic injury; (3) Studies with splenectomy also underwent distal pancreatectomy; (4) Studies with splenectomy or spleen-preserving splenic hilar dissection induced by enlarged nodes at splenic hilar were excluded; (5) Studies with distal gastric cancer (barely metastasize to splenic hilar lymph node). (6) Studies did not distinguish the G + S and G + SPSHD.

### Data extraction and quality assessment

After removal of duplicates, two investigators (Z.G and G.Y) independently screened all titles and abstracts for eligibility. Any discrepancies were resolved by consensus and arbitration by a panel of other investigators (W.F and L.S) within the review team. Only studies published in full text were included. Experimental studies in animal models, single case reports, technical reports, reviews, abstracts, editorials and studies in languages other than English were excluded after review. If a trial was covered in more than one reports we used a hierarchy of data sources.

Two investigators (Z.G and X.G) independently reviewed the main reports and supplementary materials, extracted the relevant information from the included trials with a predefined data extraction sheet. Extracted data included study characteristics, baseline patient characteristics, intervention details and outcome measures. Risk of bias assessment was conducted by 2 authors in duplicate (G.M and L.X) using the Newcastle-Ottawa Scale (NOS) system. The maximum possible score is 9 points, and NOS scores greater than six are considered indicative of high-quality studies [17].

### Statistical analysis

The network meta-analysis (NMA) which considers direct and indirect evidence on the benefits and harms among multiple treatments simultaneously, done in line with the items of the Preferred Reporting Items for Systematic Reviews and Meta-Analyses for Network Meta-Analyses (PRISMA-NMA) [18]. Details of statistical approaches applied in this study are provided as follow: The network geometry was used to graphically summarized the relationship of the three treatments (G-A, G + S and G + SPSHD), including both direct and indirect comparison. STATA in this network framework, the information of treatment G + S vs. G-A and G + S vs. G + SPSHD could be extracted directly from the publications. The existing network relationship enables us to construct the indirect comparisons (G-A vs. G + SPSHD) from two trials that have one treatment in common (G + S), and through network meta-analysis to get pooled estimated effects of both direct and indirect treatments. We performed this process using GeMTC (Generate Mixed Treatment Comparisons) package of R (version 3.4.3) through calling the code of Winbugs (version 1.4.3, [19]. The Winbugs is a software based on Bayesian Markov chain Monte Carlo which fully preserves randomised treatment comparisons within trials [20–22]. Furthermore, trace plots and the Brooks-Gelman-Rubin [23] statistic were assessed to ensure convergence (Appendix 1).

As measures of the primary treatment, the odds ratio (OR) and the standardized mean difference (SMD) was respectively calculated for dichotomous outcomes and continuous data, both with its 95% confidence interval (CI). The study endpoint was overall survival rate and the hazard ratio (HR) was assessed for the treatment effects. The HR with 95% CI were obtained directly from ready-made data of the included publications if at all possible. Otherwise, the parameter were estimated through extracting survival information from Kaplan-Meier curves using Engauge Digitizer version 4.1 (free software downloaded from http://sourceforge.net) and converting the survival rate into HRs described by Parmar et al. [24]. Estimates of relative treatment effects was plotted via forest plots and that of rank probabilities was plotted using the rank plot, a rank plot created using the rankogram function from the gemtc R package visually illustrating probabilities that each treatment is ranked. Above each treatment, the numbers of columns for all treatments in a network relationship is corresponding in a rank plot. The height of the column represents the magnitude of the ranking probability, and the column color from dark to light represents a sort order (1st to last). The higher the ranking, the more recommended the treatment [25]. A common between study heterogeneity parameter I-squared was assumed for all comparisons using ‘mtc.anohe’ method of the GeMTC R package. While the inconsistency for a comparison could not be assessed for the reason that the direct and indirect comparison did not co-exist in any branch of the comparison. (version 3.2.2). Heterogeneity was regard as low or high for I-squared values < 25% or > 75%, respectively [26]. Two-tailed *P* values of 0 .05 were used for statistical significance.

## Results

### Treatment strategy network

**Fig. 1:**
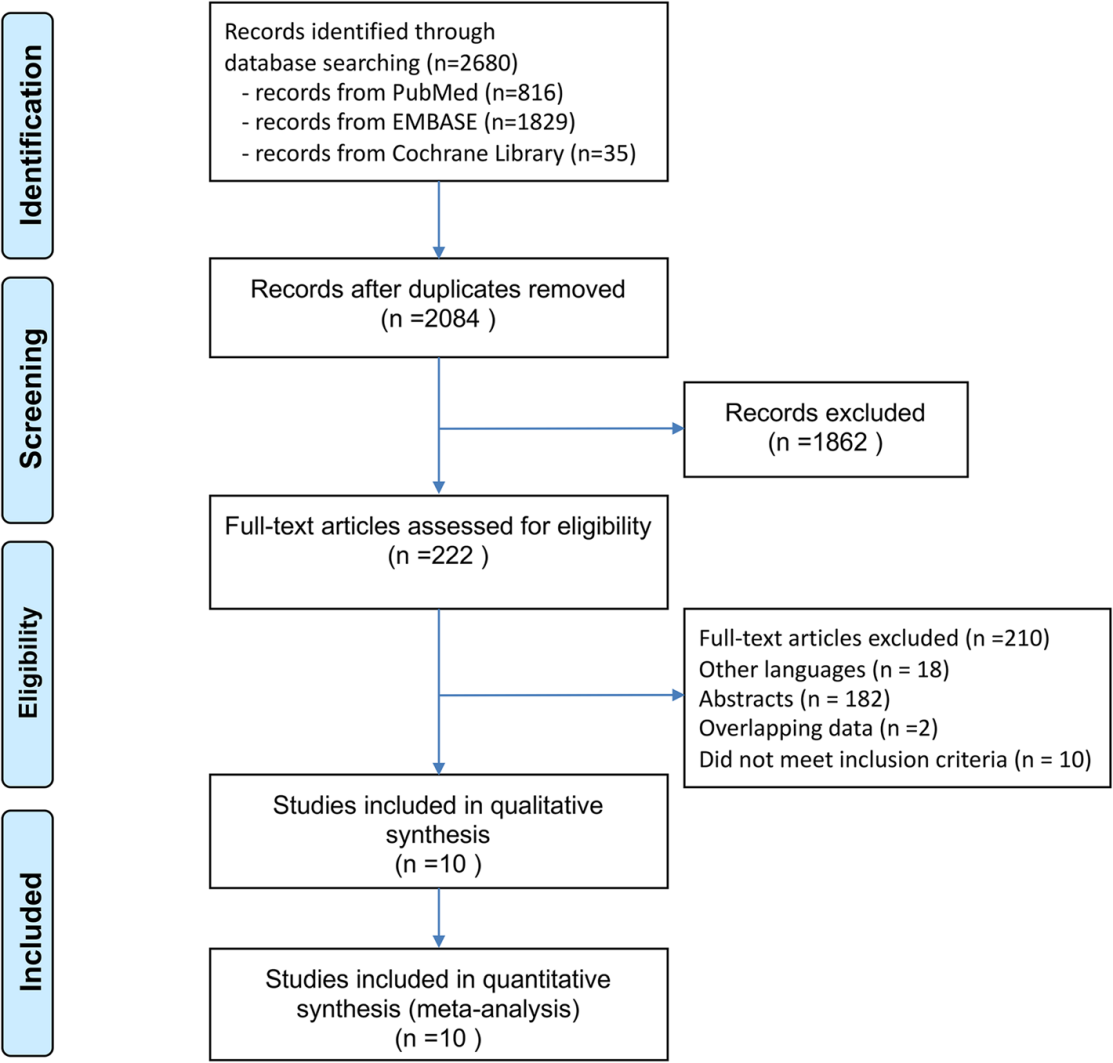
PRISMA flow chart for the study

Overall, 2530 citations were identified by the search and 222 potentially eligible articles were retrieved in full text (Fig. 1). Finally, 10 studies involving 2565 patients which containing any 2 of the 3 surgical procedures (G-A, G + S and G + SPSHD) were included in the analyses [14, 27–35]. The study characteristics of the publications included in the meta-analysis were shown in Table 1.

**Table 1:** Characteristics of included studies

Author	Year of Publication	Country	Group	No of patients	Tumor Location	Quality score
Erturk	2003	Turkey	G-A G + S	23 38	Upper, Middle	7
Ji	2016	China	G-A G + S	243,105	Upper	8
Lee	2001	Korea	G-A G + S	173,492	Upper, Middle, Whole	8
Kodera	1997	Japan	G-A G + S	57,129	Upper	7
Ohkura	2017	Japan	G-A G + S	45 63	Upper	7
Yao	2011	China	G-A G + S	61 51	Upper, Whole	7
Fang	2012	Taiwan	G + SPSHD G + S	68 47	Upper	7
Kwon	1997	Korea	G + SPSHD G + S	232,260	Upper, Middle, Whole	8
Oh	2009	Korea	G + SPSHD G + S	267 99	Upper	8
Son	2017	Korea	G + SPSHD G + S	68 44	Upper, Middle, Whole	7

**Fig. 2:**
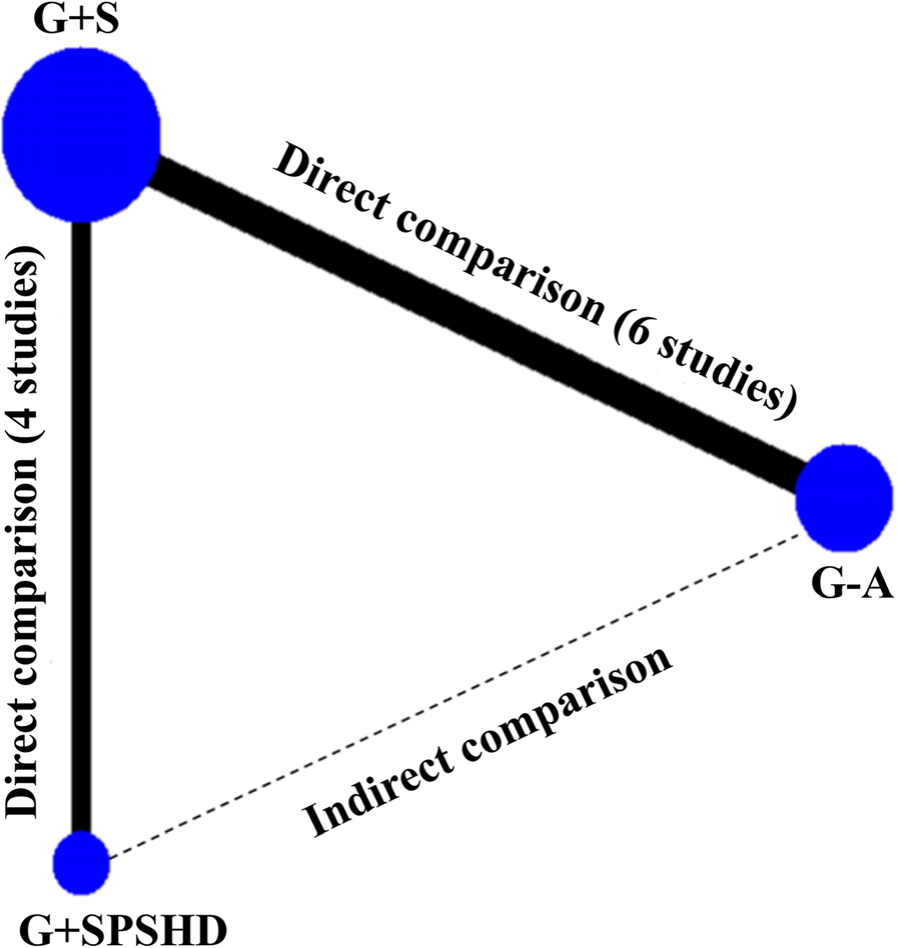
Network of eligible comparisons for efficacy

The network analysis diagram was shown in Fig. 2. Direct meta-analysis was feasible for the following comparisons: G-A versus G + S (6 trials, *n* = 1480), and G + S versus G + SPSHD (4 trials, *n* = 1085). However, G-A with G + SPSHD could only be compared through indirect meta-analysis.

### Risk of bias assessment

Two independent reviewers (G.M and L.X) evaluated the quality of evidence reported in each study using the Cochrane risk of bias tool. A summary of the risk of bias for each included study is shown in Table 1. All ten articles were scored≥7, which ensured the high quality of the included articles.

### The pooled result of perioperative complications

**Fig. 3:**
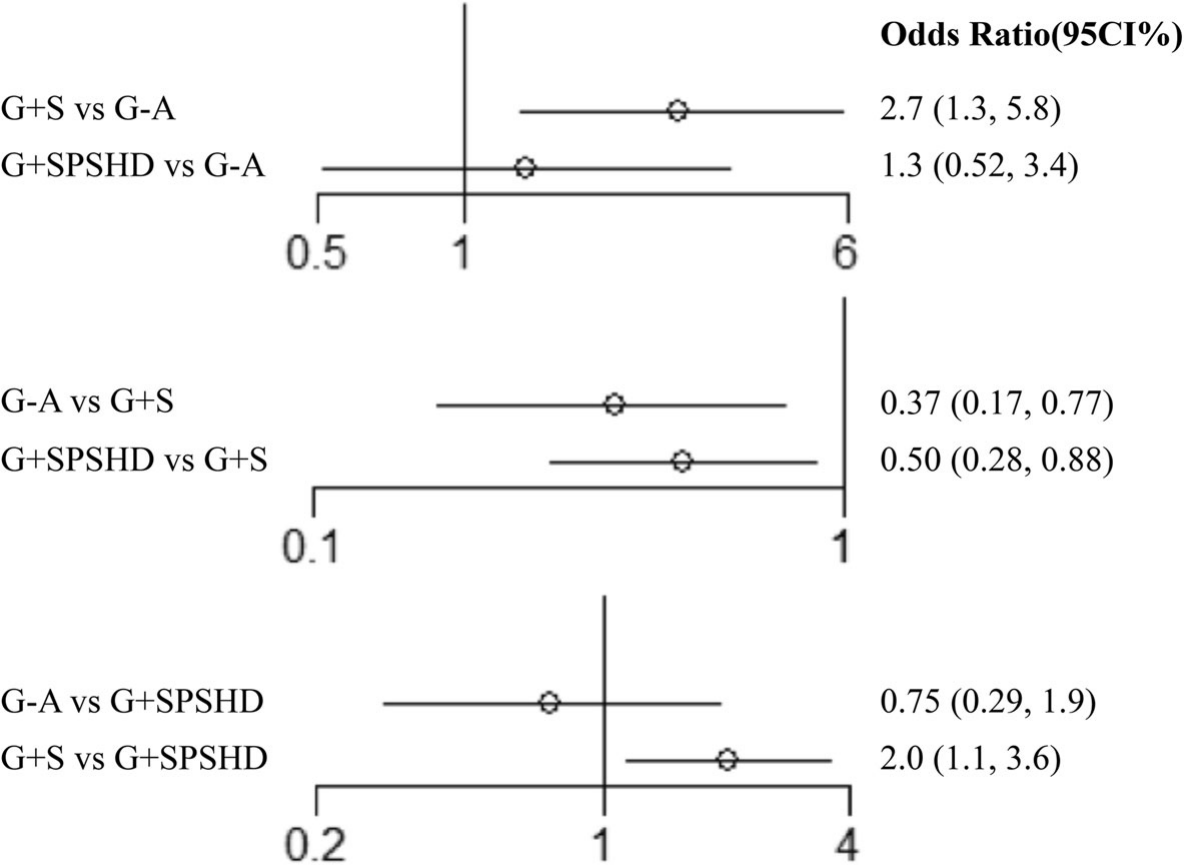
The pooled result of perioperative complications

The most common complications were anastomotic leakage, pancreas-related complications, bleeding, wound complications, pulmonary and ileus. The results of the network meta-analyses for the perioperative complications was presented in Fig. 3. In the direct comparison analyses, both G-A (OR: 0.37, 95%CI: 0.17–0.77) and G + SPSHD group (OR: 0.50, 95%CI: 0.28–0.88) showed lower total complication rate compared with G + S group. The indirect comparison showed that the total complication rate of G + SPSHD group did not differ significant from that of G-A group (OR: 1.3, 95%CI: 0.52, 3.4).

### The network meta-analyses of the 5-year overall survival rate

**Fig. 4:**
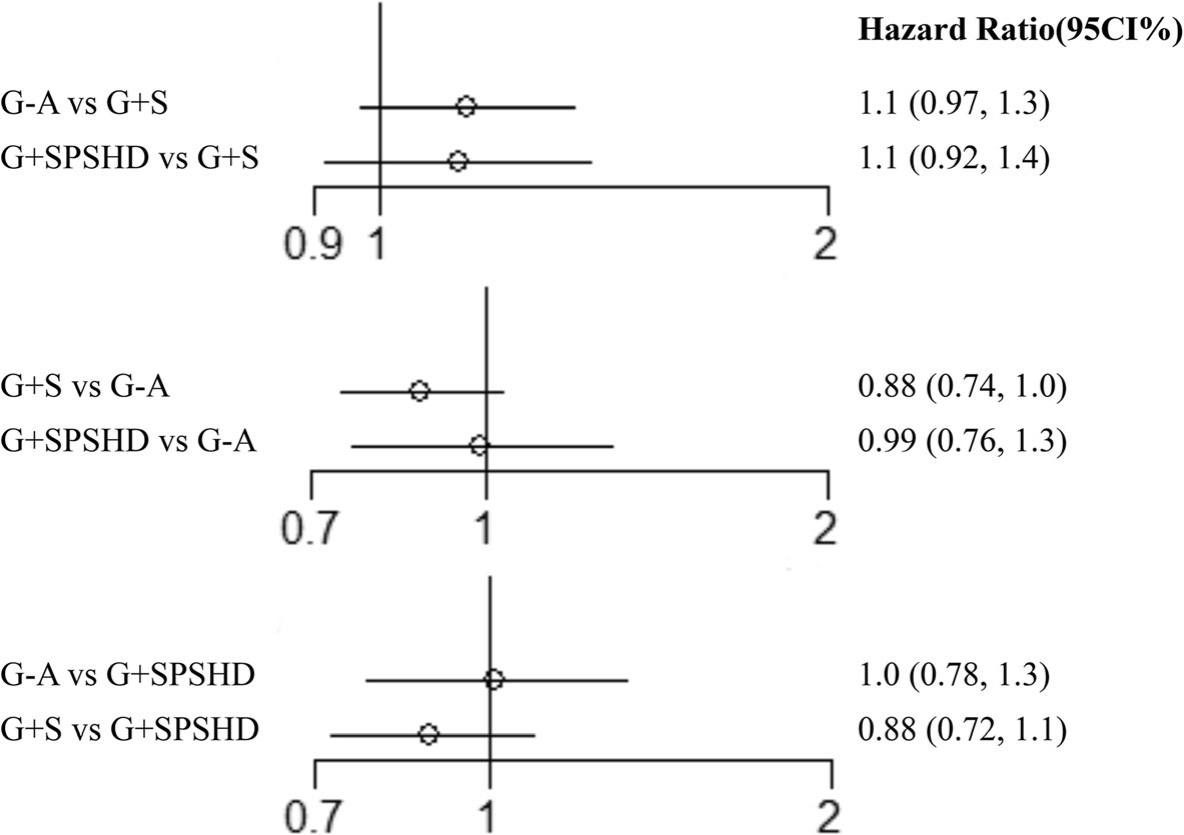
The network meta-analyses of the 5-year overall survival rate

As HR in one study [29] was not shown and could not be extracted from survival curves according to the Parmar’s method [24], only 9 studies were included for survival analysis. The results from direct and indirect meta-analysis of overall survival rates were shown in forest plot (Fig. 4). In the direct comparison analyses, both G-A (HR: 1.1, 95%CI: 0.97–1.3) and G + SPSHD group (HR: 1.1, 95%CI: 0.92–1.4) showed no significant difference compared with G + S group in 5-year overall survival rates. The indirect comparison result showed that the prognosis was also comparable between G-A and G + SPSHD group (HR: 1.0, 95%CI: 0.78–1.3).

### Ranking plots of treatments based on probabilities

**Fig. 5:**
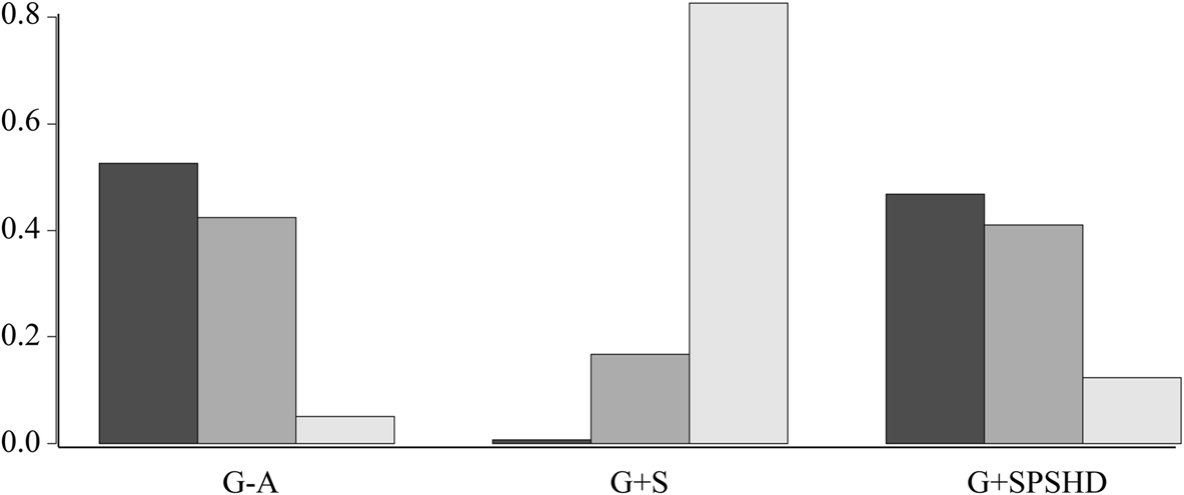
Barplots for the ranking probabilities of each treatment

Similar to the above trend, the rank plot showed that the G-A group is the optimal intervention because it has the highest probability of being ranked first, followed by the G + SPSHD group and the last one is the G + S group (Fig. 5).

## Discussion

The spleen is rarely a target of direct invasion by gastric cancers, but sometimes LNM is found in splenic hilus. For that reason, prophylactic removal of the splenic hilar lymph nodes in gastric cancer has been advocated by some investigators. However, it remains inconclusive whether prophylactic clearance of lymph node station No. 10 is associated with overall survival in patients undergoing resection of gastric cancer. Thus, the present study aims to investigate the impact of prophylactic No.10 lymph node clearance on the perioperative complications and prognosis of middle and upper third gastric cancer. This is the first analysis to compare the impact of G-A, G + S and G + SPSHD on the perioperative complications and prognosis of middle and upper third gastric cancer. Our findings indicated that prophylactic No.10 lymph node clearance was not recommended for treatment of upper and middle third gastric cancer.

To date, three prospective randomized controlled trials (RCTs) have evaluated the impact of prophylactic splenectomy on prognosis in patients with gastric cancer. The Csendes trial [36] enrolling 187 patients in a single institution showed that splenectomy has no effect on survival after total gastrectomy (42% vs 36%). The Japanese trial [37] enrolling 505 patients in multicenter showed no survival difference between G + S group and G-A group (75.1% vs 76.4%) in upper third gastric cancer. Another small-scale trial [38] enrolling 79 patients reported by Toge et al. showed a slightly better 5-year survival with splenectomy, but the difference was not statistically significant either. In our present study, splenectomy is not recommended for gastric cancer patients without direct invasion of the splenic hilar lymph nodes because this procedure does not increase the survival rate, but only increase the postoperative complications.

Many studies have assessed the role of prophylactic splenectomy in gastric cancer patients with high risk factors for No.10 lymph node metastasis. Ohno et al. suggested that G + S is the optimal procedure in proximal T3 gastric cancer [9]. However, Ito et al. reported that, in patients with pT3–4 tumors, prophylactic splenectomy has no significant survival benefit [39]. Furthermore, studies show that there was no significant difference in recurrence rate and 5-year survival rate at stage III and IV the patients who underwent total gastrectomy with or without splenectomy [8, 14, 29, 40]. With respect to tumor location, Ohkura et al. found that prophylactic splenectomy has no significant prognostic impact compared with G-A in patients with tumor involving the greater curvature [32]. It is worth mentioning that the inclusion criteria of those studies were not rigorous in selecting patients. As a result, the conclusions of these studies should be explained with cautious. Thus, large well-designed studies are needed to explore the role of No.10 lymph node clearance in patients with possible splenic hilar lymph node metastasis in the future.

It is a matter of debate whether the spleen should be preserved or removed in prophylactic splenic hilar lymph node dissection. Supporters of splenectomy argued that G + S could facilitate dissection of lymph nodes at the splenic hilum and along the splenic artery more radically, while others thought that G + SPSHD was quite enough for splenic hilar lymph node dissection. Moreover, splenectomy has been reported to be associated with increased morbidity and mortality rates due to the importance of the spleen as a part of the immune system [16, 41]. The Korean RCTs [42] enrolling 207 patients showed slightly but not significantly better survival in G + S group than G + SPSHD group (54.8% vs 48.8%). Therefore, the study suggested that prophylactic lymphadenectomy with splenectomy was not justified, and spleen-preserved lymphadenectomy might be a better option for advanced upper and middle third gastric cancer patients. In our current study, G + S also has no advantages in prophylactic splenic hilar lymph node dissection compared with G + SPSHD.

Study comparing the prognosis between G-A group and G + SPSHD group was limited. A retrospective study by Yang et al. reported that there was no significant difference of 5-year survival rates between the two groups [43]. Another study by Bian et al. also found that G + SPSHD could not improve the overall survival compared with G-A in patients with advanced proximal gastric cancer without metastasis to No. 4 s lymph node. Meanwhile, G-A group had better short-term outcomes, faster recovery, and lower postoperative morbidity rates than G + SPSHD group [44]. However, the two studies involved a few patients with gross invasion of the splenic hilum, so we excluded them in our present research. In our indirect comparison meta-analyses, the total complication rate and 5-year survival rate of G + SPSHD group did not differ significant from that of G-A group. However, according to the results of cumulative ranking probability plots, the G-A has highest probability to be optimal surgical procedure for patients with gastric cancer. What’s more, with respect to the safety, the fragile texture of the spleen and large amount of vessel branches being located at the splenic hilum may increase the risk of No. 10 lymphadenectomy.

There was only one meta-analysis which based on three RCTs evaluated the impact of splenectomy on long-term survival of patients with gastric cancer in the literature [45]. Yang et al. concluded that splenectomy did not show a beneficial effect on survival rates compared to splenic preservation. However, in their meta-analysis study, they failed to make a distinction between the G-A group and G + SPSHD group, and classified them as spleen-preserving group.

There are several limitations in our present study. First, the investigations enrolled in our network meta-analysis were all retrospective studies which introduces a possible limitation of selection bias, detection bias, and performance of analysis bias. Second, we focused on overall survival only and did not analyze progression-free survival. This was partly due to literature limitations, as some studies did not report progression-free survival for one or both groups. Third, according to our inclusion criteria, the tumors were not strictly limited to upper third of the stomach.

## Conclusion

This network meta-analysis systemically reviewed currently available evidence for treatment of gastric cancer in the second-line setting to address the knowledge gap regarding the optimal extent of lymphadenectomy for these patients. Through both direct and indirect comparisons, we demonstrated that prophylactic G + S and G + SPSHD were not recommended for treatment of middle and upper third gastric cancer.

## Additional file

**Additional file 1.** Assessment of trace plots and the Brooks-Gelman-Rubin statistic.

## Data Availability

Please contact the author Hongwei Zhang (zhenghwfmmu@126.com) upon reasonable requests.

## Abbreviations

CI: Confidence interval
G + S: Gastrectomy combination with splenectomy
G + SPSHD: Gastrectomy combination with spleen-preserving splenic hilar dissection
G-A: Gastrectomy alone
HR: Hazard ratio
LNM: Lymph node metastasis
NOS: Newcastle-Ottawa Scale
OR: Odds ratio
PRISMA-NMA: Preferred Reporting Items for Systematic Reviews and Meta-Analyses for Network Meta-Analyses
RCT: Randomized controlled trial
SHLNM: Splenic hilar lymph node metastasis
SMD: Standardized mean difference
SPSHD: Splenectomy or spleen-preserving splenic hilar dissection
