# Supplementary figures and images for: Necessity of prophylactic splenic hilum lymph node clearance for middle and upper third gastric cancer: a network meta-analysis
Source: BMC Cancer. 2020 Feb 24;20:149. doi: 10.1186/s12885-020-6619-8 (PMC7041100; doi:10.1186/s12885-020-6619-8)

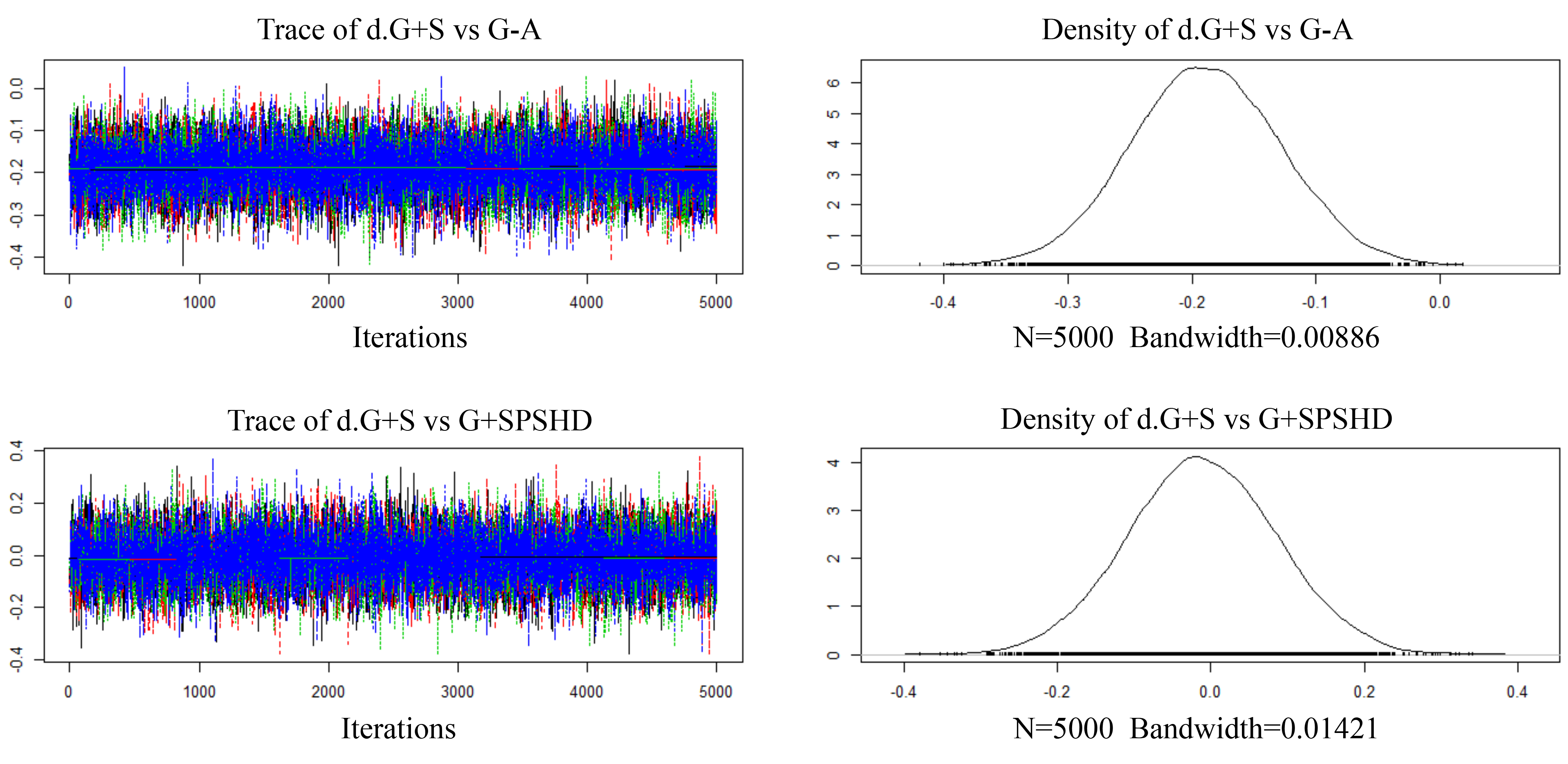

Supplement: Supplementary file 1 — Additional file 1. Assessment of trace plots and the Brooks-Gelman-Rubin statistic. [file 12885_2020_6619_MOESM1_ESM.tif]
